# *Thymus syriacus* Essential Oil Extract: Potential Antileishmanial Activity Induced by an Apoptotic-like Death

**DOI:** 10.3390/antibiotics14030293

**Published:** 2025-03-12

**Authors:** Basem Battah, Teresa Chianese, Luigi Rosati, Giacomo Petretto, Chadi Soukkarieh, Marco Ferrari, Vittorio Mazzarello, Aleksandra Barac, Aleksandar Peric, Matthew Gavino Donadu

**Affiliations:** 1 Department of Microbiology and Biochemistry, Antioch Syrian Private University, Maaret Saidnaya 22734, Syria; basem.battah.sc@hotmail.com; 2 Department of Biology, University Federico II, Via Cintia 21, 80126 Napoli, Italy; teresa.chianese2@unina.it (T.C.); luigi.rosati@unina.it (L.R.); 3 Department of Medicine, Surgery and Pharmacy, University of Sassari, 07100 Sassari, Italy; gpetret-to@uniss.it; 4 Faculty of Science, Damascus University, Damascus 30621, Syria; soukkarieh@gmail.com; 5 Department of Biomedical Sciences, University of Sassari, 07100 Sassari, Italy; dr.marcoferrari@gmail.com (M.F.); vmazza@uniss.it (V.M.); 6 Clinic for Infectious and Tropical Diseases, University Clinical Center of Serbia, 11000 Belgrade, Serbia; 7 Faculty of Medicine, University of Belgrade, 11000 Belgrade, Serbia; 8 Department of Otorhinolaryngology, Faculty of Medicine of the Military Medical Academy, University of Defense, 11042 Belgrade, Serbia; aleksandarperic1971@gmail.com; 9 Scuola di Specializzazione in Farmacia Ospedaliera, Department of Medicine, Surgery and Pharmacy, University of Sassari, 07100 Sassari, Italy; mdonadu@uniss.it; 10 Hospital Pharmacy, Giovanni Paolo II Hospital, ASL Gallura, 07026 Olbia, Italy

**Keywords:** *Thymus syriacus*, antileishmanial, apoptosis, essential oil

## Abstract

**Background:** Chemotherapy continues to be the cornerstone for the management of leishmaniasis. The preferred medications are pricey and have a number of unfavorable side effects. These restrictions make it necessary to produce novel antileishmanial chemicals, and plants have opportunities in this respect. **Objectives:** This study aimed to evaluate the antileishmanial properties of *Thymus syriacus* essential oil and its mechanisms of action. **Results:** Our findings demonstrated that *Thymus syriacus* essential oil, rich in thymol, exhibited potent antileishmanial activity, with an IC50 value of approximately 1 µg/mL against *L. tropica* promastigotes. Furthermore, the cell cycle arrest at the sub-G0-G1 phase supported the theory that the leishmanicidal effect was mediated by apoptosis. **Methods:** The essential oil was characterized using gas chromatography–tandem mass spectrometry. Antileishmanial activity against *L. tropica* promastigotes was assessed, with mechanisms confirmed via flow cytometry. **Conclusions:** These results confirm the potential of *Thymus syriacus* essential oil as a promising therapeutic candidate for the treatment of leishmaniasis.

## 1. Introduction

Leishmaniasis is a parasitic disease caused by several protozoal species of the *Leishmania* genus. This illness is brought on by an intracellular parasite. The type of parasite involved, its host and vector, the disease’s incidence rate, and its mortality all affect the disease’s manifestations. A female sand fly transmits the infection during blood meals [1]. Over 90% of cases of cutaneous leishmaniasis (CL) are reported in Afghanistan, Saudi Arabia, Algeria, Brazil, Iran, Iraq, Syria, and Sudan. Reports of the visceral form originate primarily from Brazil, Sudan, and India. In Iran, more than half of the cases of CL increased [2] in 20 out of 32 provinces, highlighting a growing public health concern [3].

The scarcity of effective antileishmanial medications, their high cost, prolonged treatment duration, and the emergence of drug resistance underscore the urgent need for alternative therapies. Pentavalent antimony remains the first-line treatment, but its efficacy has declined due to resistance, while second-line drugs like amphotericin B and pentamidine are highly toxic [4]. The absence of an effective vaccine for disease prevention further exacerbates this challenge. Consequently, a systematic approach has been established to screen natural products, including plant-based compounds, for potential antileishmanial activity [5].

Essential oils, recognized for their antibacterial and antifungal properties, are promising candidates. For instance, essential oils extracted from *Achillea millefolium* and *Ocimum basilicum* have shown detrimental effects on trypanosomes, a protozoal species closely related to *Leishmania* [6]. The World Health Organization (WHO) advocates for the use of traditional medicine, especially in regions with limited access to healthcare, making plants a critical source of bioactive compounds [7]. Several herbal plants traditionally used in medicine, such as *Rosmarinus officinalis*, *Lavandula angustifolia*, and *Lavandula xintermedia*, exhibit antiparasitic effects, further supporting the exploration of plant-based treatments for leishmaniasis [8,9].

Among these, *Thymus syriacus*, a plant native to the Middle East, has demonstrated strong antifungal, antibacterial, and antibiofilm properties in previous studies. However, its antiparasitic properties, particularly its antileishmanial activity, remain underexplored. Given its rich phenolic composition, dominated by thymol, *Thymus syriacus* is hypothesized to exhibit significant antileishmanial effects. This study addresses a critical gap in the literature by investigating the antileishmanial properties of *Thymus syriacus* essential oil and elucidating the mechanisms underlying its apoptotic-like effects [10,11].

## 2. Results

### 2.1. Thymus syriacus Essential Oil Extraction and Chemical Characteristics

Qualitative chemical characterization of the essential oil was carried out by GC analysis coupled with a mass spectrometer detector, while semi-quantitative determinations were obtained by GC analysis coupled with a flame ionization detector. As reported in Table 1, 24 main compounds were identified, including both hydrocarbons (22.5%) and oxygenated terpenoids (73.6%). The chemical composition was dominated by carvacrol, which accounted for over 68% of the total compounds, followed by Fp-cymene (8.7%) and γ-terpinene, which accounted for 6.3%. The carvacrol isomer, thymol l, accounted for 2.7%. This composition underscores the predominance of thymol, a compound linked to potent antimicrobial and antiparasitic properties.

### 2.2. Thymus syriacus Essential Oil Cellular Viability

**Figure 1:**
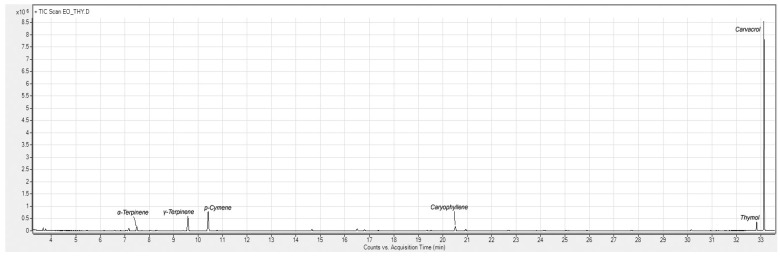
Gas chromatogram figure of *Thymus syriacus* essential oil.

**Figure 2:**
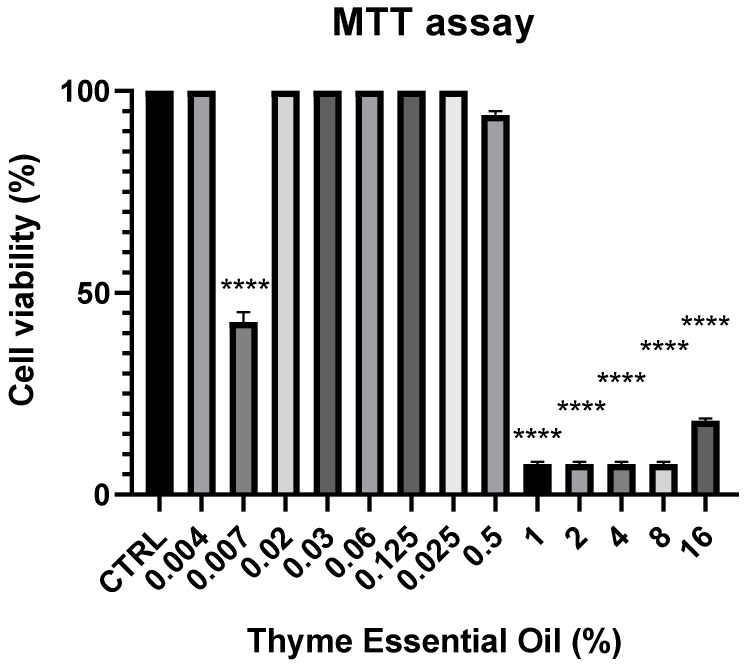
Summary graph of the MTT viability assay against *Thymus syriacus* essential oil in the PNT1a cell line. Different concentrations of *Thymus syriacus* essential oil used for the assay: 16%, 8%, 4%, 2%, 1%, 0.5%, 0.25%, 0.125%, 0.062%, 0.031%, 0.015%, 0.007%, and 0.004%. CTRL (control: non-treated cells). Significant differences in cell viability are evident between those treated with the oil, particularly with the highest and lowest doses of essential oil compared to the control (CTRL) (untreated group) (**** *p* < 0.0001).

The MTT cell viability test used to evaluate the effects of *Thymus syriacus* essential oil on normal prostate cells (PNT1a) showed that at high concentrations (16% to 1%), after 24 h of treatment, cell viability was not optimal; in fact, as the oil concentration decreased, cell viability increased. Finally, cell viability was best at a concentration of 0.06% (Figure 1). The percentage viability data demonstrated no significant cytotoxicity at concentrations below 0.06%, making it a viable therapeutic candidate. The viability percentage was calculated according to the following formula: (OD [570 nm] evaluated sample/(OD [570 nm] negative control) = R; R × 100 = % cell viability (Figure 2).

### 2.3. Juli-Stage Inverted Microscope Cell Visualization

**Figure 3:**
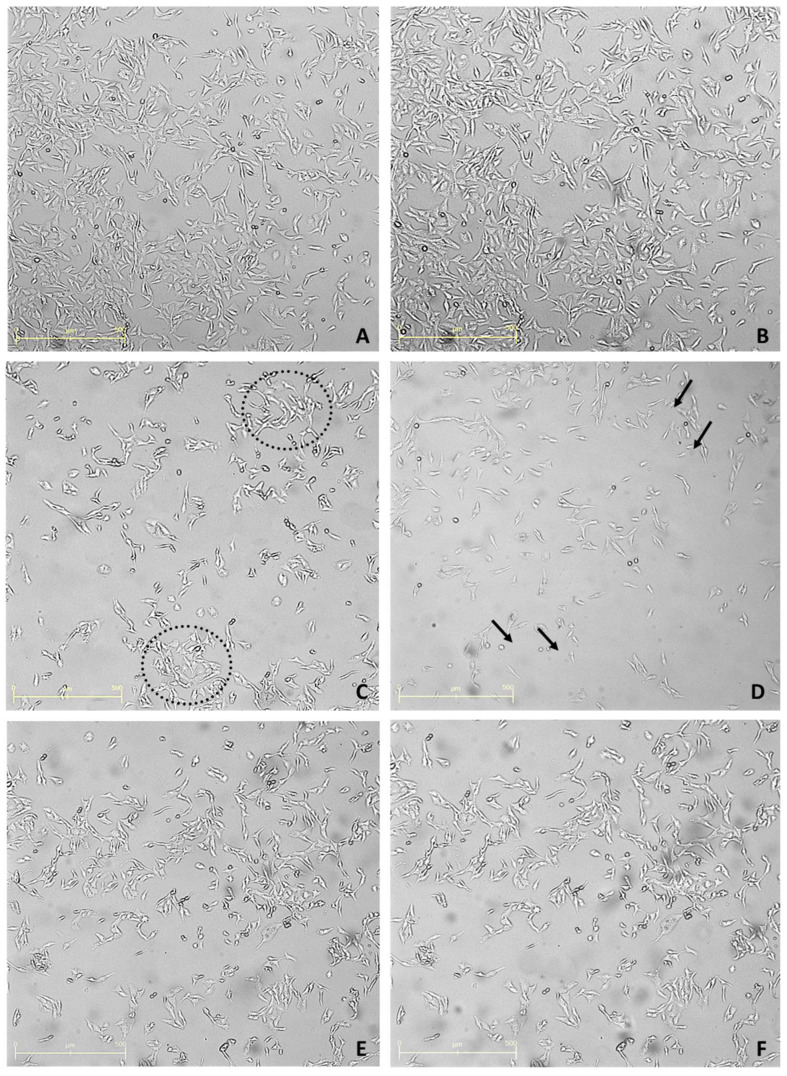
**Images of PNT1a at time 0 and 24 h after treatment with *Thymus syriacus* Essential Oil.** (**A**): Control at time zero. (**B**): Control after 24 h. (**C**): Cells treated with the concentration of *Thymus syriacus* Essential Oil (16%) at time zero. (**D**): Cells treated with the concentration of *Thymus syriacus* Essential Oil (16%) after 24 h. (**E**): Cells treated with the concentration of *Thymus syriacus* Essential Oil corresponding to the viability from the MTT assay (0.06%) time zero. (**F**): Cells treated with the 0.06% concentration of Thyme after 24 h. Dotted circles: high confluence zone. Arrows: area where no cells are evident after treatment. Scale bars correspond to 500 μm in all figures.

To support the data obtained from the MTT viability test, images were taken with the aid of an inverted microscope (JuLI™ Stage Real-Time CHR, NanoEnTek Inc., Hwaseong-si, Republic of Korea) equipped with a camera. This allowed the real-time monitoring of cells under optimal conditions within the incubator. Images were taken at time zero and after 24 h for three conditions: untreated cells (Figure 3A,B), cells treated with 16% essential oil (Figure 3C,D), and cells exposed to 0.06% essential oil (Figure 3E,F). The highest and lowest treatment concentrations, corresponding to the highest and lowest cell viability, were analyzed. Microscopic analysis confirmed the MTT findings, with a marked reduction in cell number at 16% concentration and no visible changes in cells treated with 0.06% essential oil.

### 2.4. Antileishmanial Effect

**Figure 4:**
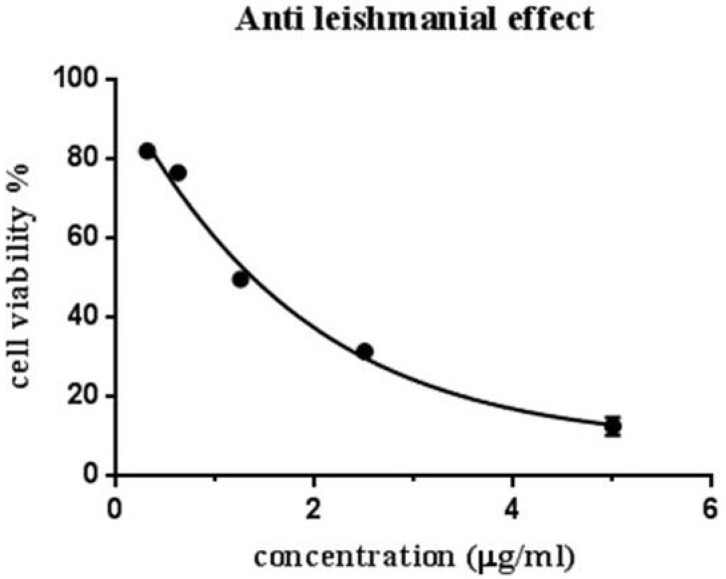
Antileishmanial activity of *Thymus syriacus* essential oil. The viability was calculated after incubation of promastigotes for 24 h with serial dilution of *T. syriacus* essential oil at different concentrations (0.31, 0.62, 1.25, 2.5, 5 µL/mL). The data presented represent a single experiment replicated three times.

In this study, promastigotes of L. tropica were incubated with various concentrations of essential oil (0.31, 0.62, 1.25, 2.5, 5 µL/mL), and cell viability was determined after 72 h using the MTT assay. The findings demonstrated that essential oil *T. syriacus* exhibited potent antileishmanial activity against the promastigote forms in a dose-dependent manner (*p* < 0.05). Our findings reveal that *Thymus syriacus* essential oil exhibits potent antileishmanial activity, with an IC50 value of approximately 1 µg/mL. These results are presented graphically in Figure 4.

### 2.5. Apoptosis-Like Death in Leishmania

**Figure 5:**
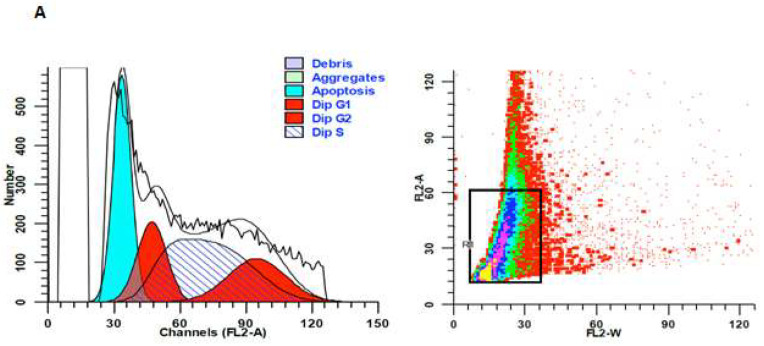

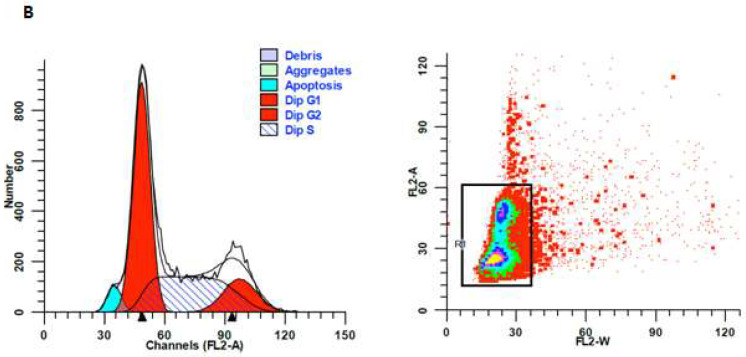
Analysis of cell cycle status of *Thymus syriacus* essential oil-treated promastigotes. (**A**) represents the treated group with IC50 ≈ 1 µg/mL of *Thymus syriacus* essential oil for 18 h after annexin V-FITC/PI dual staining. (**B**) represents the non-treated (control) group. Dip G1 (diploid G1 phase), Dip G2 (diploid G2 phase), Dip S (diploid S phase). R1 indicates apoptotic cell death.

Promastigote forms of *L. tropica* were treated with the IC50 concentration of *Thymus syriacus* essential oil for 18 h, and cellular death was determined after annexin V-FITC/PI dual staining by flow cytometry (Figure 5). Approximately 29% of *L. tropica* promastigotes underwent apoptosis (early + late apoptosis), compared to 4.8% in the untreated control. This indicates that apoptosis plays a significant role in the antileishmanial effect of the essential oil.

## 3. Discussion

Research has demonstrated that several traditionally used medicinal herbs exhibit antileishmanial properties, supporting their usage in traditional medicine [12]. Plant essential oils and active ingredients can be utilized in place of or in addition to existing antiparasitic treatments [13]. Essential oils have recently been shown to have leishmanicidal activity [14]. The present investigation provides novel evidence of the antileishmanial efficacy of *Thymus syriacus* essential oil against *L. tropica*, with a notable IC50 value of approximately 1 µg/mL. The findings confirm its potent activity, particularly through an apoptotic mechanism [15]. This study disclosed that the cytotoxicity and chemical composition of *Thymus syriacus* essential oil, primarily thymol, contribute significantly to its antileishmanial activity.

The observed apoptotic activity suggests a cellular mechanism of action that aligns with the activity of other thymol-rich essential oils reported in the literature [16]. For instance, previous studies on thymol derivatives, such as benzoyl-thymol, demonstrated enhanced efficacy against Leishmania species compared to thymol alone or amphotericin B, further underscoring the potential of this compound. The chemical variability of essential oils, influenced by harvest season and geographic origin, plays a crucial role in their biological activity [11]. The previous literature on the essential oil extracted from Thymus syriacus shows a slight variability in chemical composition. Jamil et al. (2010) identified thymol as the main compound of the essential oil (74%), followed by carvacrol (9%). Tayoub et al. (2024) compared three Thymus species and found Thymus syriacus to be the richest in thymol, which accounted for over 77% of the total compounds. By contrast, Tubmen et al. (1994) studied a chemotype rich in carvacrol, with a chemical composition characterized by high amounts of carvacrol (15.87%) and thymol (49.04%) [17,18,19]. In this study, thymol constituted 2.68% of the oil and carvacrol 68.36%, contrasting with levels reported in previous research, which highlights its potential as a bioactive compound in contrast to previous research on Thymus syriacus bioss (40%) and Thymus vulgrais with (47%) thymol [20]. In addition, another study described carvacrol as the main compound, with thymol (3.6–4.3%), carvacrol (33.4–39.5%), carhyophyllene (5.3–6.9%), and p-cymene (3.9–4.7%) [21]. In other research, the distribution of Thymus syriacus essential oil components were carvacrol (34.49%), dihydrocarvone (5.17%), β-caryophyllene (5.32%), p-cymene (4.45%), farnesol (5.26%), limonine (2.68%), menthol (3.55%), myrecene (3.68%), γ-terpinene (7.44%), terpinene-4-ol (3.20%), and thymol (3.82%) [22].

Furthermore, it has been demonstrated in multiple investigations that thymol and its derivatives, along with related compounds such as carvacrol and γ-terpinene, possess strong antibacterial, antifungal, and antiparasitic properties against specific pathogenic strains [18]. These findings highlight the potential for utilizing Thymus syriacus essential oil in pharmaceutical or nutraceutical applications, given its potent apoptotic effects and inhibitory action. Additionally, the findings corroborate earlier studies that demonstrated the effectiveness of essential oils derived from Thymus species, with thymol identified as a key bioactive component [19,23].

A critical aspect of this study is the apparent discrepancy between antioxidant activity and antileishmanial efficacy [24,25,26]. Although high phenolic content, including thymol, typically correlates with strong antioxidant properties, the exact link between these attributes and the observed apoptotic-like effects warrants further exploration. It is plausible that thymol’s activity may involve interactions with specific cellular pathways or membrane functions, contributing to apoptosis independently of its antioxidant capacity. This highlights the need for future research to unravel the molecular mechanisms underpinning these effects [27,28].

A key strength of this work was to test the antimicrobial effect of *T. syriacus* on both human and Leishmania cells. This is important for several scientific and applicative reasons. In particular, it allows selectivity to be assessed, as comparing cytotoxicity in human cells helps determine the therapeutic margin, i.e., the effective dose against the pathogen without harmful effects on the host. Low selectivity could indicate systemic toxicity, making the compound unsuitable for therapeutic use and necessitating further studies.

The potential applications of *Thymus syriacus* essential oil extend beyond its standalone use. Future investigations should focus on exploring its synergistic effects with existing antileishmanial drugs, as such combinations may enhance efficacy while mitigating resistance development. Furthermore, efforts to develop a stable dosage form, such as tablets or emulsions, could facilitate its clinical application [29,30].

Overall, this study provides a foundation for the development of *Thymus syriacus* essential oil as a novel antileishmanial treatment. Continued research is necessary to validate these findings in vivo and optimize its use in pharmaceutical settings, potentially addressing the pressing need for safer and more effective therapies for leishmaniasis.

## 4. Materials and Methods

### 4.1. Essential Oil Preparations

The *Thymus syriacus* samples were collected in May from rural areas of Damascus during the blossoming stage. The gathered aerial components were dried for one week at a temperature of 25 °C in a shaded area to preserve their bioactive compounds. According to established protocols, steam distillation was used to extract the essential oil using a Clevenger-style apparatus. The resulting isotropic mixture of water and oil was separated using chloroform (non-polar solvent, Merck^®^, Boston, MA, USA), followed by rotary evaporation to extract the essential oil. The extraction yielded an oil concentration of 1.5% (*w*/*v*) from the dried leaves of *Thymus syriacus* [9,11,31].

### 4.2. Chemical Characterization of Essential Oil

The GC analysis of the EO was carried out using an Agilent 4890N instrument equipped with an FID as well as on an Agilent 6890 GC and coupled with an Agilent 5973 MSD detector. The chromatographic separations were performed on an HP-5 column 30 m 0.25 mm i.d., 0.5 μm film thickness column (Agilent, Santa Clara, CA, USA) as well as on a ZB-WAX column 30 m ID 0.25 mm, 0.25 μm film thickness (Phenomenex, Torrance, CA, USA). The following temperature program was used for the HP5 column: 50 °C was held for 3 min, then increased to 210 °C at a rate of 4 °C/min, held for 15 min, then increased at a rate of 10 °C/min up to 300 °C, and finally maintained for 15 min. The following temperature program was used on the ZB-WAX column: 40 °C hold for 4 min, then increased to 150 °C at a rate of 5 °C/min, held for 3 min, then increased to 240 °C at a rate of 10 °C/min, and finally held for 12 min. Helium was used as the carrier gas at a constant flow of 1 mL/min. The data were analyzed using a Mass Hunter Workstation B.06.00 SP1, (Agilent, Santa Clara, CA, USA) and identification of the individual components (Table 1) was performed by comparison against co-injected pure compounds and by matching the MS fragmentation patterns and retention indexes using the built-in libraries, literature data, or commercial mass spectral libraries (NIST/EPA/NIH 2008; HP1607 purchased from Agilent Technologies). A hydrocarbon mixture from C8–C23 was injected under the same conditions in both columns to obtain the linear retention indexes. The results in Table 1 are reported as relative percentages obtained by the internal normalization of the FID chromatogram without considering any response factor. α-Pinene, p-Cymene, Terpinen-4-ol, Borneol, and Caryophyllene oxide were purchased from Sigma-Aldrich. α-Phellandrene, Limonene, γ-Terpinene, Linalool, Caryophyllene <E>, α-Humulene, and α-Terpineol were purchased from Fluka Chemika.

### 4.3. Cell Culture

PNT1a human prostate cells were cultured in Roswell Park Memorial Institute (RPMI) medium (Sigma-Aldrich^®^, Saint Louis, MO, USA) supplemented with 10% fetal bovine serum (FBS, Sigma), 1% L-glutamine, and 2% penicillin/streptomycin in an incubator maintained at 37 °C with 5% CO2 and controlled humidity. Cells were detached enzymatically using 0.25% trypsin/EDTA upon reaching 70% confluence. This setup ensured optimal growth conditions for subsequent viability and cytotoxicity assays [32]. The cell line used was PNT1a, and this is the only description of the cells we can provide: PNT1a cells (human prostate cell line derived from immortalizing adult prostate epithelial cells) were obtained from the E.C.A.C.C. (European Collection of Cell Culture, Salisbury, UK).

#### MTT Vitality Test on PNT1a Cells

The cytotoxic effects of *Thymus syriacus* essential oil on PNT1a cells were assessed using the MTT assay [31]. Cells were seeded at a density of 5000 cells/well in 96-well plates containing 100 µL of complete RPMI culture medium and allowed to adhere for 24 h. Cells were counted using the Luna II automated cell counter (Biosystem, Atlanta, GA, USA). After medium replacement with RPMI containing 1% FBS, cells were exposed to various concentrations of *Thymus syriacus* essential oil diluted in culture medium with 0.5% Tween 80. Dilutions ranging from 16% to 0.004% were evaluated in triplicate. Following 24 h of incubation, MTT reagent (5 mg/mL) was added to each well for 4 h, after which the medium was aspirated and replaced with 100 µL of DMSO to solubilize the formazan crystals. Absorbance at 570 nm was measured using a Synergy HTX multi-mode microplate reader. Viability was calculated as a percentage relative to the untreated control group, with a threshold of 50% viability indicating cytotoxicity.

### 4.4. Parasites

A local strain of wild-type *Leishmania tropica*, identified via nested-PCR, was used. Promastigotes were cultured in RPMI-1640 medium supplemented with 10% FBS, 50 U/mL penicillin/streptomycin, and 2 mM/L glutamine. Cultures were maintained at 26 °C, and parasite growth was monitored via phase-contrast microscopy. This ensured a stable model for antileishmanial testing [20,33].

### 4.5. MTT Leishmanial Cell Assay

Promastigotes were seeded at a density of 1 × 10^6^ cells/well in 96-well plates and treated with various concentrations of *Thymus syriacus* essential oil (0.31–5 µL/mL). After 24 h of incubation, cell viability was assessed using the MTT assay, following the manufacturer’s instructions (Sigma-Aldrich^®^). Absorbance at 570 nm was recorded, and viability was expressed as a percentage relative to untreated controls. Mean absorbance values were used to generate dose–response curves, with IC50 values calculated from the data.

### 4.6. Cell Cycle Distribution

Leishmania promastigotes were exposed to the IC50 concentration of *Thymus syriacus* essential oil for 24 h. Cells were centrifuged at 300 g for 5 min, washed with PBS, and stained with 125 µg/mL propidium iodide (PI) and 0.1 mg/mL RNAase. Samples were analyzed using a FACSCalibur flow cytometer (BD Life Sciencies, Franklin Lakes, NJ, USA) to determine cell cycle phase distribution.

### 4.7. Apoptosis Status

Promastigotes treated with *Thymus syriacus* essential oil at the IC50 concentration for 24 h were washed in PBS and resuspended in binding buffer. Annexin V-FITC and PI staining were performed according to the manufacturer’s protocol (BD Pharmingen™, San Diego, CA, USA). Stained cells were analyzed using flow cytometry to distinguish early and late apoptosis from necrosis.

### 4.8. Statistical Analysis

All experiments were conducted in triplicate. Data are presented as means ± standard deviation (SD). Statistical analysis was performed using GraphPad Prism 6 software (GraphPad Software Inc., La Jolla, CA, USA). One-way or two-way ANOVA, followed by post hoc corrections, was applied to assess differences, with significance set at *p* < 0.05.

## 5. Conclusions

This study demonstrates the potential of *Thymus syriacus* essential oil as a novel antileishmanial agent, showcasing significant apoptotic-like effects against *Leishmania tropica* promastigotes. The essential oil, characterized by a high thymol content (75%), exhibited a potent IC50 value of approximately 1 µg/mL, highlighting its efficacy in vitro. The results suggest that apoptosis serves as a primary mechanism of the oil’s antileishmanial activity, offering a promising alternative to conventional treatments. Despite these encouraging findings, further research is necessary to establish the clinical applicability of *Thymus syriacus* essential oil. Future studies should focus on validating its efficacy in in vivo models, exploring potential synergistic effects with existing antileishmanial drugs, and developing optimized dosage forms such as tablets or emulsions. Additionally, assessing the safety and pharmacokinetic properties of the essential oil will be crucial to advancing its therapeutic use.

In conclusion, *Thymus syriacus* essential oil represents a promising natural candidate for the development of safer, cost-effective, and effective treatments for leishmaniasis, particularly in regions where drug resistance and toxicity of conventional treatments pose significant challenges.

## Figures and Tables

**Table 1:** Main compounds detected in thymus essential oil. Results were expressed as relative percentages obtained by FID peak area normalization. RI_(WAX)_ retention index calculated on a ZB-WAX column, RI_(HP5)_: retention index calculated on an HP5 column. ID: Identification method: RI refers to calculated retention indices compared to those reported in the NIST Chemistry WebBook database and the Adams book database. MS indicates comparison of the fragmentation pattern with available databases, and STD refers to pure standard co-injection. RI lit wax: literature retention index from the NIST Chemistry database; RI lit HP5 literature kovats index from R.P. Adams.

Compound	A%	RI_(WAX)_	RI_(HP5)_	ID	RI Lit Wax	RI Lit HP5
α-Pinene	0.99	1031	931	RI, MS, STD	1024	939
α-Thujene	0.66	1035	923	RI, MS	1038	930
β-Pinene	0.17	1102	975	RI, MS	1096	979
2-Carene	0.16	1140	1004	RI, MS	1146	1002
α-Phellandrene	0.23	1157	992	RI, MS, STD	1158	1003
α-Terpinene	1.58	1172	1015	RI, MS	1167	1017
Limonene	0.31	1191	1036	RI, MS, STD	1192	1029
β-Phellandrene	0.26	1200	1030	RI, MS	1212	1030
γ-Terpinene	6.30	1238	1057	RI, MS, STD	1223	1060
p-Cymene	8.71	1263	1023	RI, MS, STD	1252	1025
α-terpinolene	0.17	1274	1087	RI, MS	1278	1089
*cis*-Sabinene hydrate	0.58	1459	1071	RI, MS	1444	1070
*trans*-Sabinene hydrate	0.32	1520	1090	RI, MS	1488	1098
Linalool	0.21	1548	1105	RI, MS, STD	1552	1097
Caryophyllene <E>	2.44	1580	1421	RI, MS, STD	1580	1429
Terpinen-4-ol	0.72	1594	1187	RI, MS, STD	1599	1177
α-Humulene	0.20	1646	1456	RI, MS, STD	1660	1455
α-Terpineol	0.16	1688	1189	RI, MS, STD	1684	1189
Borneol	0.18	1690	1169	RI, MS, STD	1698	1169
β-Bisabolene	0.22	1719	1502	RI, MS	1736	1506
Cubenene	0.14	1749	1513	RI, MS	1778	1523
Caryophyllene oxide	0.42	2069	1592	RI, MS, STD	2023	1583
Thymol	2.68	2193	1307	RI, MS, STD	2198	1352
Carvacrol	68.36	2223	1298	RI, MS	2239	1299

## Data Availability

Data are contained within the article.
